# Time to rethink the reported disease-modifying treatment effects on cognitive outcomes: Methods and interpretative caveats

**DOI:** 10.3389/fneur.2022.995690

**Published:** 2022-09-01

**Authors:** Andrés Labiano-Fontcuberta, Enric Monreal, Julián Benito-León

**Affiliations:** ^1^Department of Neurology, University Hospital12 de Octubre, Madrid, Spain; ^2^Department of Neurology, University Hospital Ramón y Cajal, Universidad de Alcalá, Ramón y Cajal Institute for Health Research (IRYCIS), Spanish Network of Multiple Sclerosis (REEM), Madrid, Spain; ^3^Centro de Investigación Biomédica en Red Sobre Enfermedades Neurodegenerativas (CIBERNED), Madrid, Spain; ^4^Department of Medicine, Complutense University, Madrid, Spain

**Keywords:** information processing, cognitive dysfunction, randomized controlled (clinical) trial, multiple sclerosis, practice effect

## Introduction

The literature on the effect of multiple sclerosis disease-modifying treatments (DMTs) on multiple sclerosis-related cognitive dysfunction has grown exponentially over the last few years. A detailed analysis of this topic can be found in the comprehensive systematic review and meta-analysis of Landmeyer et al. (1). Although this review rightly highlighted important weaknesses of the literature reviewed, it was not able to delve into some aspects of the field that should not be overlooked and deserve much more in-depth reflection.

For instance, some research shows that DMTs greatly benefit processing speed outcomes (Table 1). As an example of how significant these effects can be, 60% of alemtuzumab-treated patients had clinically meaningful improvement in SDMT score just after the second course of the treatment (2), and 62.2% of ocrelizumab-treated patients achieved clinically meaningful improvements over 96 weeks, a percentage that was even higher (72%) in those patients with mild impairment at baseline (3). We consider that these results, along with other aspects detailed throughout this opinion article, should be questioned from the viewpoint of biological plausibility and should make us rethink whether the current research regarding the effects of DMTs on longitudinal cognitive performance is adequate.

**Table 1:** Unreasonable results of studies analyzing the effects of disease-modifying treatments on cognitive outcomes.

**Disease-modifying treatments**	**Study design**	**Follow-up time**	**Cognitive assessment**	**Outstanding results**
Alemtuzumab (2)	Observational Single-arm	15 months	Digit span forward TMT-A, TMT-B RAVLT, RCFT SDMT Verbal fluency words	The proportion of patients impaired in processing speed performance decreased from 50 to 20% 60% of alemtuzumab-treated patients achieved clinically meaningful improvement
Ocrelizumab (3)	Pooled analysis of the OPERA I and OPERA II trials interferon-beta 1a-controlled	22 months	SDMT PASAT	66.2% of ocrelizumab-treated patients achieved clinically meaningful improvement, and this percentage is even higher (72%) among patients with mild impairment at baseline
Dimethyl fumarate (4)	Observational single-arm	24 months	BRB battery^*^ stroop test	37.2% of patients improved their cognitive impairment index
Siponimod (5)	*Post-hoc* analysis of the EXPAND trial placebo-controlled	24 months	BVMT-R PASAT SDMT	34.9% of the SPMS patients in the Siponimod group had sustained cognitive improvement over 24 months, but also a surprisingly high percentage (27%) in the placebo group PASAT and BVMT-R scores did not differ significantly between Siponimod and placebo
Ozanimod (6)	Phase III SUNBEAM trial interferon beta 1a-controlled	12-month	SDMT	Patients who achieved clinically meaningful improvement (slightly greater in the ozanimod group) steadily increased in both treatment groups
Natalizumab (7)	ASCEND trial placebo-controlled SPMS patients	24-month	SDMT	Cognitive improvement increased from 50% at 12 weeks to more than 70% at 84 weeks in the placebo group.

The articles with the most outstanding results have been selected for this table. It is possible that not all potentially relevant articles have been included.

BRB, brief Repeatable battery; BVMT-R, brief visuospatial memory test-revised; PASAT, Paced Auditory Serial Addition Test; TMT-A, Trail Making Test A; TMT-B, Trail Making Test B; RAVLT, Rey auditory verbal learning test; RCFT, Rey complex figure test; SDMT, Symbol Digit Modalities Test; SPMS, Secondary progressive multiple sclerosis.

* Tests included: SRT-LTS (Selective Reminding Test-Long-Term Storage); SRT-CLTR (Selective Reminding Test-Consistent Long-Term Retrieval); SRT-D (Selective Reminding Test-Delayed); SPART and SPART-D (10/36 Spatial Recall Test and delayed); PASAT 3; PASAT 2; SDMT.

This is not a comprehensive review of the cognitive assessment literature's methodological weaknesses, which can be found elsewhere (1), but instead a wake-up call that provides researchers and trialists with controversial findings that merit much further reflection before moving the research forward.

## Concerning results that deserve closer attention and criticism

### Information processing speed outcomes steadily increase during follow-up

The mean change from baseline in processing speed scores has consistently been higher at month 6, and, after that, it steadily improves in successive evolutions (2–12), The best example is the observation reported by Koch et al. (7), who found that SDMT scores steadily increased overall 28 testing sessions throughout the 2 years of follow-up in the ASCEND trial in secondary progressive multiple sclerosis (SPMS), Similarly, Morrow et al. (10) reported average SDMT baseline scores of 46.8 and an average final score of 62.2 at week 48 (an average improvement of 32.9% over baseline) in 660 natalizumab-treated patients with MS; and Woelfle et al. (11) observed an average improvement of 25.4% correct responses over baseline with electronic SMDT. The effect is not mitigated by using *Z*-scores, as evidenced by our results in which the percentage of patients who underwent cognitive improvement at 12 months (defined by an increase of 0.5 standard deviations) was twice as frequent as those who experienced cognitive worsening (26.6 vs. 11.4%) (9).

The impact of the practice effect on the improved SDMT performance is so large that a simple change in the order of the key symbols has been shown to completely reverse the increase in SDMT scores and returned it to baseline values (12).

Although this improvement has been chiefly considered short-term learning- effect as most of it occurred up to the third repetition, there was still a significant improvement from the third repetition onward (11, 12). For instance, Roar et al. (12) reported that the SDMT performance improved by 1.2 points/test during the first 6 months and by 0.4 points/test thereafter.

Even more concerning is that this practice effect seems more significant than the reported between-group mean difference in longitudinal cognitive outcomes, which raises questions about whether the differences observed in favor of some treatments are meaningful. A secondary analysis of the EXPAND trial of siponimod in SPMS reported a between-group mean difference in SDMT scores of 2.3 points between baseline and 24 months in favor of siponimod compared to placebo (5), and a pooled analysis of the OPERA, I, and OPERA II studies reported a between-group difference in SDMT scores of 1.3 points in favor of ocrelizumab compared to interferon beta1a (3). Although these differences are statistically significant, they seem futile compared to the observed magnitude of SDMT score change related to practice effects.

### Too high a percentage of patients, including those with progressive phenotypes, experience clinically meaningful cognitive improvement during follow-up

When using the proposed four-point definition of clinically meaningful change, patients are much more likely to experience cognitive improvement than worsening throughout their longitudinal cognitive assessment.

The most unexpected result has been observed in SPMS patients, in whom cognitive improvement increased from around 50% of participants at 12 weeks to more than 70% at 84 weeks, including those in the placebo group (7). In the *post hoc* EXPAND trial, 27% of the SPMS patients in the placebo group had sustained cognitive improvement over 24 months (5), which does not fit with the expected relentless decline of cognitive function that many people with SPMS are supposed to experience. As a whole, more than half of the patients regardless of the DMT received, have been reported to show a *clinically meaningful change* in their cognitive performance in just a few months, which we consider to be utterly unreasonable. Table 1 summarizes some of the main astonishing results.

## Discussion

### Cognitive tests practice effects need to be addressed in much more detail

While the field researchers are well aware of the practice effects challenges, it is concerning that the studies aimed to assess the effects of DMTs on cognitive evolution have systematically overlooked the practice effects in interpreting their results. For example, the SDMT practice effect is wholly disregarded in the OPERA and the SUNBEAM studies (3, 6). Furthermore, these studies expedite and amplify the learning effects by using short and frequent retesting intervals (at 12-week intervals in the OPERA study (3) and 6-month intervals in the EXPAND (5) and SUNBEAM (6) studies), which contribute to exacerbating the improved SDMT performance.

First, given that without a comparator group is impossible to disentangle whether longitudinal cognitive performance improvement represents practice or treatment effects, the meaningfulness of the uncontrolled observational studies, which represent the majority of the published literature (1), should be questioned.

Regarding randomized controlled trials, it is mandatory to precisely define which approaches have been used to control for practice effect. The following methodological proposal could partially mitigate the practice effect and help strengthen the effect of a given DMT on the cognitive evolution: (i) Assess longitudinal performances also in randomized clinical control groups; (ii) Use adequate retesting intervals by performing longitudinal cognitive tests less frequently (i.e., at 12-month intervals); (iii) Discard the results from the first cognitive performances. If the frequency of SDMT testing is conducted monthly or at 6-month intervals, as in clinical trials, the results from the first 2–3 cognitive performances should be discarded. This approach would not be necessary if annual cognitive examinations were applied. Regardless of the frequency of the SDMT administration, we firmly believe that the first ever cognitive evaluation should be removed, and therefore the second cognitive measurement (providing the cognitive assessment is not conducted at monthly intervals) could be accepted as the baseline cognitive evaluation on which to assess the DMT effects on longitudinal cognitive performance; (iv) Apply parallel or alternate forms of SDMT which have shown excellent reliability (13). Regarding this last point, it should be stressed that although it appears that practice effects are modestly mitigated by using alternating forms (13), the reality is that to date, no study has been specifically designed to determine to what extent the use of alternate forms decreases the learning effect compared to the continued use of the same SDMT key. Since electronic tests [e.g., the iPad®-based Processing Speed Test (PST)] randomly generate a new key for each new administration (14), we believe it would be particularly interesting to assess the extent to which the learning effect is mitigated by comparing the rearranged key-based electronic tests with the paper-and-pencil SDMT in both MS patients and controls. Until these differences are adequately defined through specifically designed studies, we believe we should be cautious in asserting that using SDMT alternate forms can *per se* prevent the learning effects-relate cognitive improvement. Indeed, one-third of untreated SPMS patients showed cognitive improvement despite using alternate SDMT forms in the EXPAND study (5).

Importantly, although processing speed is the core domain of cognitive impairment in MS, the effects of DMTs on other cognitive domains less susceptible to the learning effects remain uninvestigated. Thus, although integrating neuropsychological batteries into MS daily clinical practice remains challenging due to the need for at least 20 minutes of one-on-one testing for every patient, the inclusion in randomized controlled trials of other cognitive domains such as episodic memory seems reasonable. Noteworthy, Siponimod had an impressive benefit on SDMT scores compared to the placebo group. Still, there were no differences regarding the memory outcome measured as the BVMT-R (brief visuospatial memory test-revised) (5).

### The term “clinically meaningful” based on a 4-point change should be reconsidered

Although establishing a 4-point change in the SMDT as a benchmark for clinically meaningful change was objectively based on deterioration in vocational status predictions (15), the reality is that its systematic application in the literature has yielded irrational results, as described in point 2.2.

The strict application of such a narrow difference might imply that high baseline performers could reach their boundary sooner and, therefore, are much more vulnerable to a 4-point decrease. On the other hand, low baseline performers could have more room for improvement due to a floor effect. Indeed, if we take a closer look at the SUNBEAM results, we can see that the 12-month SDMT-worsened group was a much higher baseline performer than the 12-month SDMT-improved group (raw score: 52.3 vs. 45.1; standardized *z*-score: +0.38 vs. −0.15) (6), which means that the lower the baseline SDMT score is, the greater the opportunity to show a 4-point improvement. This analysis is of great importance, as the greater percentage of clinically meaningful improvements reported in favor of some DMTs could be due to an unadjusted imbalance in the group's proportion of baseline SDMT performance levels rather than a specific treatment effect on cognitive evolution.

These results involve high stakes, as it raises questions about the convenience of the SDMT as a longitudinal outcome measure of disease progression, at least over the 2-year follow-up of most studies. If the community research does not want longitudinal cognitive assessment to mask disease progression, a reframing of how we define cognitive improvement/ worsening is urgently needed. For instance, a decline of 8 or more raw score points, double the current threshold, has recently been described as the most helpful threshold for capturing a statistically reliable individual change on the SDMT (16).

We still prefer to use *relative* (e.g., a 10% loss of SDMT score) *or adjusted* [reduction of 0.5 standard deviations (*Z*-score)] score losses rather than raw score losses.

Moreover, based on these relative or adjusted definitions, we propose a new approach in which the information inherent in practice effects could be exploited as a new helpful outcome measure. In this approach, three different groups would be established based on the patient's SDMT learning curves: (1) the group with the expected physiological continuous SDMT improvement related to gaining practice. In a practice effect context, we consider this group represents a much more accurate definition than the term clinically meaningful improvement. (2) The group who remain cognitive unchanged, which should be interpreted with caution as these are patients who are not able to turn the benefit of practice effect into improvement, and (3) the group with cognitive worsening that would represent those patients with neurodegenerative damage that no longer have the brain reserve availability to maintain their SDMT scores despite the benefit of the practice effects.

Another approach that might offer more insight into the potential beneficial effects of DMTs on cognitive function would include stratifying by some baseline cognitive performance subgroups (e.g., cognitive impairment at baseline, patients with the highest baseline cognitive score, etc.), thus providing information on the rate of change in cognitive functioning across different groups.

Despite all these caveats, we are convinced that longitudinal cognitive assessment is a useful measure of disease progression and that DMTs, particularly high effective DMTs (9), have a protective effect on disease-related cognition. However, identifying and critiquing cognitive-related methodological limitations are paramount before moving the research forward. We strongly believe that using some easy-to-adopt measures, such as more adequate retesting intervals (at 12-month intervals) and replacing the 4-point threshold with more reasonable measures, might overcome cognitive-related methodological challenges.

## Author contributions

AL-F initiated the project. AL-F, EM, and JB-L made intellectual contributions, read, revised, and approved the final manuscript.

## Funding

JB-L is supported by the National Institutes of Health, Bethesda, MD, USA (NINDS #R01 NS39422), the European Commission (grant ICT-2011-287739, NeuroTREMOR), the Ministry of Economy and Competitiveness (grant RTC-2015-3967-1, NetMD—platform for the tracking of movement disorder), and the Spanish Health Research Agency (grants FIS PI12/01602 and FIS PI16/00451). The Spanish Health Research Agency and the Spanish Office of Science and Technology supported NEDICES. Information about collaborators and detailed funding of the NEDICES study can be found on the following webpage (https://ciberned.es/proyectos/nedices-1.html%20).

## Conflict of interest

The authors declare that the research was conducted in the absence of any commercial or financial relationships that could be construed as a potential conflict of interest.

## Publisher's note

All claims expressed in this article are solely those of the authors and do not necessarily represent those of their affiliated organizations, or those of the publisher, the editors and the reviewers. Any product that may be evaluated in this article, or claim that may be made by its manufacturer, is not guaranteed or endorsed by the publisher.
